# Treatment of canine leishmaniasis with marbofloxacin in dogs with renal disease

**DOI:** 10.1371/journal.pone.0185981

**Published:** 2017-10-05

**Authors:** Carmen Pineda, Escolastico Aguilera-Tejero, Maria C. Morales, Silvia Belinchon-Lorenzo, Luis C. Gomez-Nieto, Pablo Garcia, Julio M. Martinez-Moreno, Maria E. Rodriguez-Ortiz, Ignacio Lopez

**Affiliations:** 1 Department of Medicina y Cirugia Animal, University of Cordoba, Cordoba, Spain; 2 Maimonides Biomedical Research Institute of Cordoba (IMIBIC), Reina Sofia University Hospital, University of Cordoba, Cordoba, Spain; 3 LeishmanCeres Laboratory (GLP Compliance Certificated), Parasitology Unit, University of Extremadura, Caceres, Spain; 4 Nephrology Laboratory, Instituto de Investigacion Sanitaria Fundacion Jimenez Diaz, Madrid, Spain; Instituto Nacional de Salud Pública, MEXICO

## Abstract

Treatment of canine leishmaniasis (CanL) represents a challenge. Due to the high prevalence of renal disease associated to CanL, it is important to find an effective drug that does not damage the kidneys. Marbofloxacin has been shown to be effective and well tolerated in non-azotemic dogs with leishmaniasis. To evaluate the safety and efficacy of marbofloxacin in dogs with leishmaniasis and decreased renal function, 28 dogs suffering from leishmaniasis and chronic kidney disease (CKD) were treated with oral marbofloxacin at 2 mg/Kg/day for 28 days. During treatment dogs were assessed by performing weekly physical exams, measuring blood pressure and evaluating blood and urine parameters. Lymph node aspirations were also obtained at days 0 and 28. The global clinical score decreased significantly, from 6.2±3.4 to 4.7±3.1 (*p* = 0.0001), after treatment. Marbofloxacin also decreased parasitic load in 72% of the dogs. No significant differences in plasma creatinine, urine specific gravity, urinary concentrations of cystatin C, ferritin and urinary protein loss were detected during treatment. A transient but significant decrease in blood pressure was detected up to day 14 (from 180.1±36.6 to 166.0±32.7 mmHg; *p* = 0.016). Moreover, dogs showed a significant increase in plasma albumin concentration (from 15.0±5.2 to 16.6±3.9 g/L; *p* = 0.014) and a significant decrease in globulin concentration (from 59.0±18.1 to 54.1±18.0 g/L; *p* = 0.005). The results demonstrate that, in addition to being effective for treatment of CanL, marbofloxacin is a very safe drug in dogs with CKD and leishmaniasis.

## Introduction

Canine leishmaniasis (CanL) is a parasitic disease caused by the protozoan *Leishmania infantum* and transmitted by the bite of sandflies of the genus *Phlebotomus*. In the Mediterranean countries, where infection rates are up to 63% [1], CanL represents one of the leading causes of death in dogs [2]. CanL is an expanding disease and it has become one of the most important canine diseases imported to Central Europe [3].

The clinical presentation of CanL is variable and depends on the immune response of the host, thus the infection in dogs can range from subclinical and self-limiting to severe disease [4]. Three clinical presentations: cutaneous, mucocutaneous and visceral have been described [5]. Affected dogs commonly develop kidney disease associated with glomerular immune complex deposition [4, 6]. Kidney damage can progress to tubulointerstitial lesions and lead to renal failure [4]. Renal disease can be diagnosed in approximately 50% of dogs with leishmaniasis using laboratory tests [7]. Moreover, virtually 100% of dogs with leishmaniasis have histopathologic lesions compatible with nephropathy [8].

Treatment of CanL is a challenge because of the intracellular localization of the parasite [9]. Drugs for treatment of CanL include meglumine antimoniate, aminosidine and miltefosine [4], which are generally used in combination with allopurinol. Most of these treatments do not achieve complete cure of the disease and some of them can cause important side effects [5, 10]. Vomiting, diarrhoea, lethargy and nephrotoxicity are common during treatments with pentavalent antimonials and miltefosine [9–13]. Xanthine urolithiasis is a frequent complication after prolonged administration of allopurinol [14]. The toxicity of these treatments may be increased in dogs with decreased glomerular filtration rate [4]. In addition, dogs with renal involvement usually have a slower response to the drugs that requires longer treatments and, therefore, are more prone to develop adverse effects [15].

Considering the high prevalence of kidney disease in dogs with leishmaniasis, it would be important to find an effective drug with reduced toxicity in patients with renal disease. Recent data [16–18] suggest that marbofloxacin, a third generation fluoroquinolone, has similar effectiveness than miltefosine or glucantime to treat CanL. In addition, it has been shown that marbofloxacin does not induce changes in serum urea and creatinine in non-azotemic dogs and also has little or no gastrointestinal side effects [18].

We hypothesized that marbofloxacin may be a safe and effective drug for treatment of dogs with leishmaniasis and renal disease. To test this hypothesis, dogs with leishmaniasis and kidney disease were treated with marbofloxacin and followed in a prospective open clinical trial.

## Materials and methods

### Dogs

Thirty dogs attended at the Veterinary Teaching Hospital of the University of Cordoba (Spain) were studied. Sixteen dogs were males (53%) and 14 were females (47%). Dogs were aged 5.2±2.8 (range 1–14) years and weighed 20.1±9.3 (range 6–50) Kg. Hounds were predominant (16 dogs, 53%), followed by crossbreds (5 dogs, 17%), Boxers (5 dogs, 17%) and other companion dogs (Golden Retriever, Labrador Retriever, Pitbull, Argentinean Dogue) (4 dogs, 13%).

All the experimental procedures were approved by the Ethics Committee of the Veterinary Teaching Hospital of the University of Cordoba. An informed consent was obtained from the owner or person in charge of the dog prior to the inclusion in the study. To be accepted in the study, dogs had to meet the following inclusion criteria:

1. Show at least one clinical sign of CanL (e.g. lymphadenomegaly, weight loss, dermatologic signs) and be positive in a serologic test of CanL (Snap Leishmania Test, IDEXX Laboratories, Barcelona, Spain).
2. Show evidence of chronic kidney disease (CKD), stage 1 to 4 according to the classification criteria of the International Renal Interest Society (IRIS).
3. Be free from other coinfections that may contribute to progression of kidney disease: *Ehrlichia canis*, *Dirofilaria immitis*, *Anaplasma phagocytophilum*, *Anaplasma platys* and *Borrelia burgdorferi* (Snap 4Dx Test, IDEXX Laboratories, Barcelona, Spain).
4. Be free of urinary infection (negative urine culture).
5. Not have received any antibiotic, corticosteroid or anti-leishmanial treatment within the 60 days preceding inclusion.

Once admitted into the study, each dog underwent additional tests for the diagnosis of leishmaniasis and for evaluation of renal disease. Diagnosis of CanL was confirmed by two techniques: a) antibody evaluation by indirect immunofluorescence (IFI) performed on serum; and, b) presence of Leishmania DNA by real time polymerase chain reaction (PCR) in lymph node aspirates. The following parameters were evaluated for the diagnosis of kidney disease: plasma creatinine (reference range in our laboratory, 0.5–1.4 mg/dL); plasma urea, (reference range, 20 to 50 mg/dL); urine specific gravity (USG) (reference range >1030); urine protein to creatinine ratio (UPC), (normal range <0.5); and blood pressure (reference range: systolic blood pressure (SBP) <150 mmHg, diastolic blood pressure (DBP) <100 mmHg, mean blood pressure (MBP) <125 mmHg). In addition to these parameters recommended by IRIS to classify kidney disease, urinary concentrations of cystatin C and ferritin were measured. These parameters have recently been shown to be good markers of tubular (cystatin C) and glomerular (ferritin) damage in dogs [19–21].

### Treatment

All dogs received marbofloxacin (Marbocyl, Vetoquinol, Madrid, Spain) orally at a dose of 2 mg/Kg/day for 28 days. During the 28 days of treatment, there were isolated cases in which it was necessary to prescribe other drugs to control signs related to renal disease: antiemetics (n = 7), antacids (n = 7); and dermatologic signs: anti-seborrheic shampoos (n = 4). No drug that may affect renal function or blood pressure was prescribed. There were no dietary changes during the month of treatment.

### Follow up

All dogs were monitored before and every week during the treatment on days: 0, 7, 14, 21 and 28. At each examination point, a physical exam was performed, blood pressure was measured and blood and urine samples were obtained.

In the physical exam, dogs were assessed as previously reported [17] for: a) systemic signs (lethargy, epistaxis and lymphadenopathy), b) dermatological signs (alopecia, hyperkeratosis, seborrheic dermatitis, pyodermatitis, onychogryphosis, nodules and ulcers), c) musculo-skeletal signs (muscle atrophy, lameness and arthritis), d) gastro-intestinal signs (diarrhoea and intestinal bleeding) and e) ocular signs (conjunctivitis, keratitis and uveitis). Each clinical sign was scored according to its severity on a numerical scale from 0 to 3, as follows: 0, absent; 1, mild; 2, moderate; and 3, severe; except for lymphadenomegaly, ulcers, nodules and arthritis which were scored as 0, absent; 1, local; and 2, generalised. The sum of the clinical scores was calculated by adding the points given to each of the 18 clinical parameters at each examination time. In addition, body weight and rectal temperature were recorded at each examination.

Blood pressure was measured with an oscillometric blood pressure meter (petMAP graphic, Ramsey Medical Inc. Florida, USA), which quantifies the SBP, DBP and MBP. The size (40% of the limb perimeter) of the cuff was standardized for each dog. At all times, the cuff was placed in the same limb and measurements were always recorded by the same operator. The methodology was as follows: 5 measurements were obtained when the dog was quiet and relaxed in lateral recumbence. The higher and lower values were discarded and the mean of the three remaining measurements was calculated for each dog at each time point.

Blood samples were obtained from the jugular vein at all examination times and placed in heparin and EDTA-coated tubes. Samples were centrifuged and plasma was separated for measurement of creatinine and urea. Urine was collected by cystocentesis to perform an urinalysis that included: determination of USG by refractometry (Zuzi, Auxilab S.L., Navarra, Spain) and quantitative determination of proteinuria by calculating the UPC.

On day 0 and day 28, additional testing was performed as follows:

1. A complete haematological and plasma biochemistry panel was obtained. Haematologic parameters included: red and white blood cell count, leukocyte differential count, platelet count, haemoglobin concentration, mean corpuscular haemoglobin concentration (MCHC), mean corpuscular volume (MCV) and packed cell volume (PCV). The biochemistry panel included: alanine aminotransferase (ALT), alkaline phosphatase (ALP), albumin, total proteins, albumin to globulins (A/G) ratio, total calcium (tCa), phosphorus (P), total magnesium (tMg), sodium (Na), potasium (K), chloride (Cl), ionized calcium (iCa) and venous blood gas analysis (pH, PCO2, bicarbonate, base excess and anion gap).
2. Urine concentrations of cystatin C and ferritin were measured.
3. Lymph node aspirates were obtained for measurement of parasitic load by quantitative PCR. Lymph node aspirates were retrieved by fine-needle aspiration from the most enlarged lymph node. The aspirates were washed with 500μL of sterile PBS and stored in two aliquots of 250μL at -80°C for DNA extraction.

### Efficacy criteria

The treatment was considered effective if, between day 0 and day 28, the dog showed clinical improvement (i.e. the sum of clinical scores was lower than before treatment), and the parasitic load (lymph node quantitative PCR test findings) decreased. The treatment was classified as inefficacious if the dog did not clinically improve, or deteriorated, and/or parasitic load did not decrease.

### Blood and urine measurements

Plasma (urea, creatinine, ALT, ALP, total proteins, albumin, P, tCa and tMg) and urine (creatinine, protein and ferritin) biochemical parameters were measured by spectrophotometry (BioSystems S.A., Barcelona, Spain). Urine cystatin C was measured using an ELISA specifically designed for its measurement in canine samples (BioVendor, Brno, Czech Republic). UPC was calculated as follows:

UPC=([protein]inurine(mg/dL))/([creatinine]inurine(mg/dL))

For creatinine measurements in urine the sample was diluted (1/50) in distilled water.

Blood cell counts were obtained by flow cytometry (LaserCyteDx, IDEXX Laboratories, Barcelona, Spain). Electrolyte (Na, K, Cl, iCa) and acid-base parameters (pH, PCO2) were measured with selective electrodes (Siemens RAPIDLab 860, Germany).

### Polymerase chain reactions (PCR)

The DNA from lymph node aspirates was obtained using a DNA isolation kit (UltraClean BloodSpin DNA, MoBIO Laboratories, CA, USA) [22]. The detection and quantification of the DNA from *Leishmania infantum* was performed by real time PCR.

Reactions were carried out in 96 wells PCR plates in a final volume of 20 μL (4 μL of DNA + 16 μL of Reaction Mix), containing 20 μM of each primer (Leish1: 5’-AACTTTTCTGGTCCTCCGGGTAG-3’ and Leish2: 5’-ACCCCCAGTTTCCCGCC-3’(Stab Vida Laboratories, Caparica, Portugal)), 10 μM of TaqMan Probe (FAM-5’-AAAAATGGGTGCAGAAAT-3’-non fluorescent quencher-MGB (Applied Biosystems Laboratories, Foster City, CA, USA)) [23], and the iTaq Universal Probes Supermix (Bio-Rad Laboratories, Hercules, CA, USA). The thermal cycling profile used was one incubation step at 50°C for 2 minutes and an initial denaturation step at 95°C for 10 minutes, followed by 40 cycles of denaturation at 95°C for 15 seconds and annealing-extension at 60°C for one minute. Each amplification run contained positive and negative controls. All quantitative PCR analyses were performed in a Step One Plus Real Time PCR System (Applied Biosystems Laboratories, Foster City, CA, USA). A standard curve was obtained [24] using DNA extracted from six quantities of *Leishmania infantum* parasites (MCAN/ES/1996/BCN150, zymodeme MON-1) ranging from 50,000 to 0.5 (dilution factor x10). The threshold cycle (Ct) corresponding to the Y-intercept of each analysis (that is, the expected Ct value for the estimated quantity of 1 parasite) was used as cut-off, being positive those samples whose Ct values were ≤ Y-intercept value of each assay. The parasitic load in positive samples was extrapolated from the intercept of each Ct value in the Y axis of the standard curve.

### Statistical methods

Statistical analysis of the results was performed using a statistical software (SPSS version 15.0 for Microsoft Windows). A Kolmogorov—Smirnov test was carried out to test for normality. All data sets, except ALT, AST, pH, urine cystatin C and parasitic load passed normality testing. The effect of time on the normally distributed variables measured every week (urea, creatinine, UPC and blood pressure) was assessed by repeated measures of analysis of variance using a general linear model. To compare the means of the normally distributed variables measured at day 0 and at day 28 paired sample Student’s t-test was used. Wilcoxon analysis was used to compare means between day 0 to day 28 for non-normally distributed variables. Bonferroni correction was conducted to counteract type I error when multiple tests were used to evaluate a hypothesis. The Bonferroni adjusted *p* values for the different hypotheses related to the effects of marbofloxacin were: for clinical improvement (*p*<0.01), for parasite load (*p*<0.05), for renal function (*p*<0.008), for blood pressure (*p*<0.02), for haematology (*p*<0.01), for protein profile (*p*<0.02), for liver enzymes (*p*<0.02), for electrolyte profile (*p*<0.007) and for acid-base status (*p*<0.01).

The relationship between the main parameters was studied by Pearson correlation (*p*<0.01 was considered significant) and graphically displayed in a principal components analysis plot. Values are given as the mean ± standard deviation (SD).

## Results

One dog died in the course of the study and another one was lost for follow up because the owners changed address. As a result, 28 of the 30 selected dogs completed the 28 days of treatment with marbofloxacin. No important adverse events related to marbofloxacin treatment were observed. Mild diarrhoea was reported in two dogs. Since diarrhoea resolved with no specific treatment, the follow-up of these dogs progressed in conformity with the study protocol. Thus, the safety and efficacy of the treatment was evaluated in 28 dogs.

### Clinical signs

Before initiating treatment (day 0), dermatological and systemic signs were predominant. Onychogryphosis (71%, 20/28 dogs), seborrhoea (43%, 12/28 dogs) and hyperkeratosis (39%, 11/28 dogs) were the most common skin lesions. Lymphadenomegaly, either local or generalized, was detected in most dogs (75%, 21/28 dogs). Lethargy (32%, 9/28 dogs) was also a common systemic sign. Gastro-intestinal (21%, 6/28 dogs), musculo-skeletal (11%, 3/28 dogs) and ocular signs (4%, 1/28 dog) were less frequent. Epistaxis was found in 3/28 dogs (11%).

**Fig 1:**
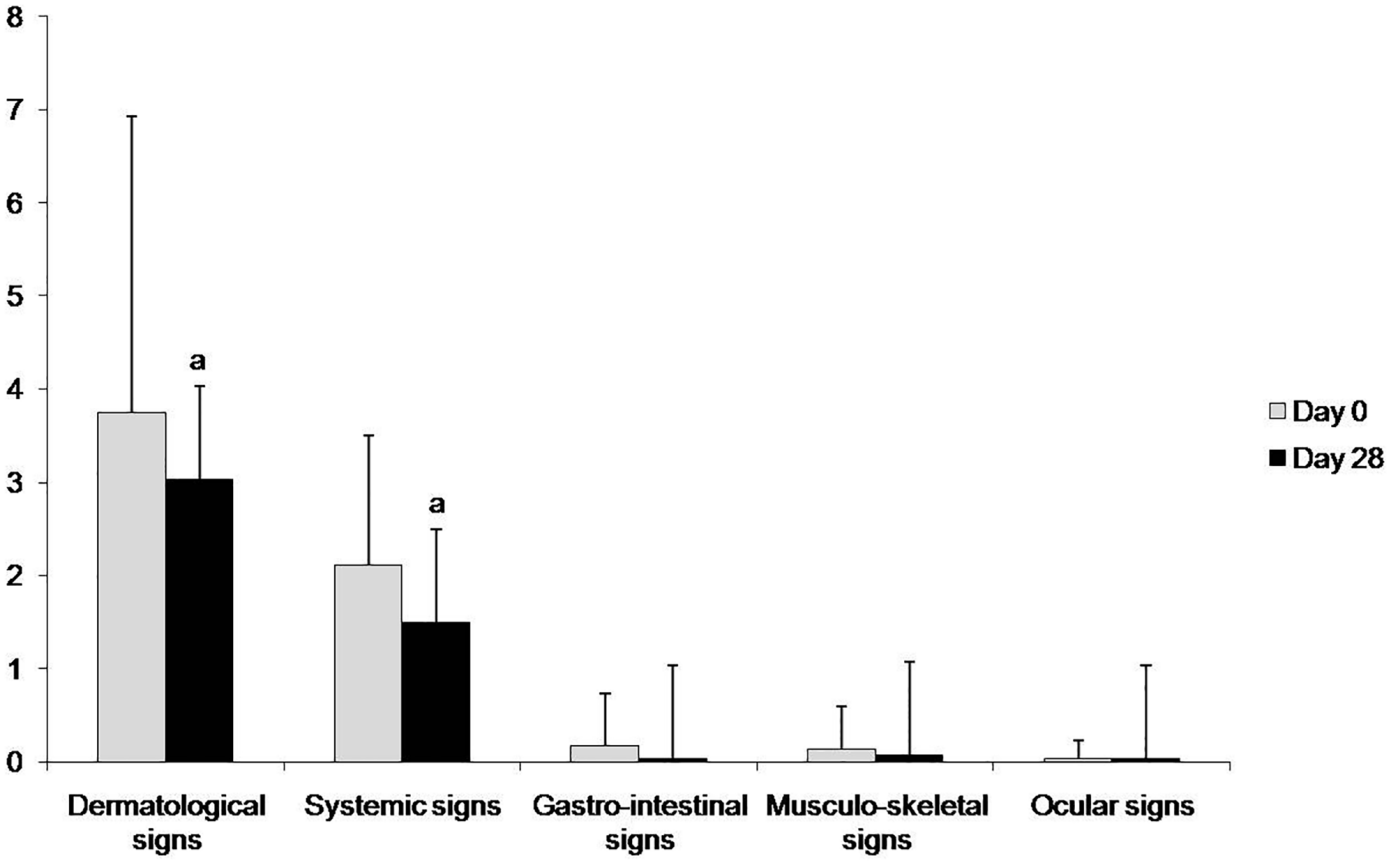
Clinical scores before (at day 0) and after (at day 28) treatment with marbofloxacin in the dogs under study. Values are expressed as the mean±standard deviation. ^a^*p*<0.01 vs day 0.

Clinical improvement was observed in 18/28 dogs (64%) after treatment (Table 1). The global clinical score decreased significantly from 6.2±3.4 to 4.7±3.1 (*p* = 0.0001) after treatment with marbofloxacin. Clinical improvement was more accentuated in dermatologic (46%, 13/28 dogs) and systemic signs (50%, 14/28 dogs). The score for dermatological signs decreased from 3.8±3.2 to 3.0±2.8, *p* = 0.008, and the score for systemic signs decreased from 2.1±1.4 to 1.5±0.9, *p* = 0.01(Fig 1). No significant changes in body weight (20.1±9.3 vs 20.5±9.0 Kg) or body temperature (38.7±0.6 vs 38.5±0.3°C) were recorded after treatment.

**Table 1:** Individual clinical score, antibody titers and parasitic load of the dogs under study before (at day 0) and after (at day 28) treatment with marbofloxacin.

Dog	Clinical score		Antibody titers	Parasitic load (parasites/μL)	
	Day 0	Day 28	Day 0	Day 0	Day 28
1	6	2	1/1280	19.4	1.4
2	4	4	1/320	80.5	5.7
3	1	2	1/320	-	-
4	5	5	1/320	5.2	19.8
5	3	1	1/320	19.4	3.2
6	8	7	1/320	1.1	71.2
7	5	3	1/640	10.5	57.0
8*	-	-	1/640	-	-
9	5	4	1/320	0.2	0.1
10	11	8	1/640	0.2	0.1
11	7	5	1/640	0.1	0.01
12	5	3	1/640	6.0	4.0
13	9	5	1/1280	9.1	0.6
14	12	11	1/640	0.3	1.6
15	4	3	1/1280	9.7	9.3
16	3	1	1/200	3.3	1.5
17	3	4	1/400	8.0	2.2
18	5	5	1/640	16.6	11.8
19	14	9	1/320	0.1	0.03
20	9	7	1/640	14.7	2.4
21	9	9	1/1280	10.7	4.4
22	6	2	1/640	49.3	42.9
23	5	5	1/320	34.3	0.03
24	3	2	1/640	-	-
25	12	13	1/320	11.3	24.2
26*	-	-	1/160	-	-
27	2	2	1/320	0.5	12.4
28	6	2	1/640	-	-
29	10	5	1/640	85.3	1.1
30	2	2	1/1280	4.2	7.5

*: dog that did not complete the study. The results obtained from these two dogs were not included in any statistical analysis. Antibody titers: normal values are considered from absence of title to 1/40; values greater than 1/160 are considered seropositive.

There are no statistical correlations between the parameters shown: at day 0: clinical score and antibody titers: r = 0.030, p = 0.884; clinical score and parasitic load: r = -0.090, p = 0.667; at day 28: clinical score and antibody titers: r = -0.076, p = 0.708; clinical score and parasitic load: r = -0.190, p = 0.364.

### Renal function

**Fig 2:**
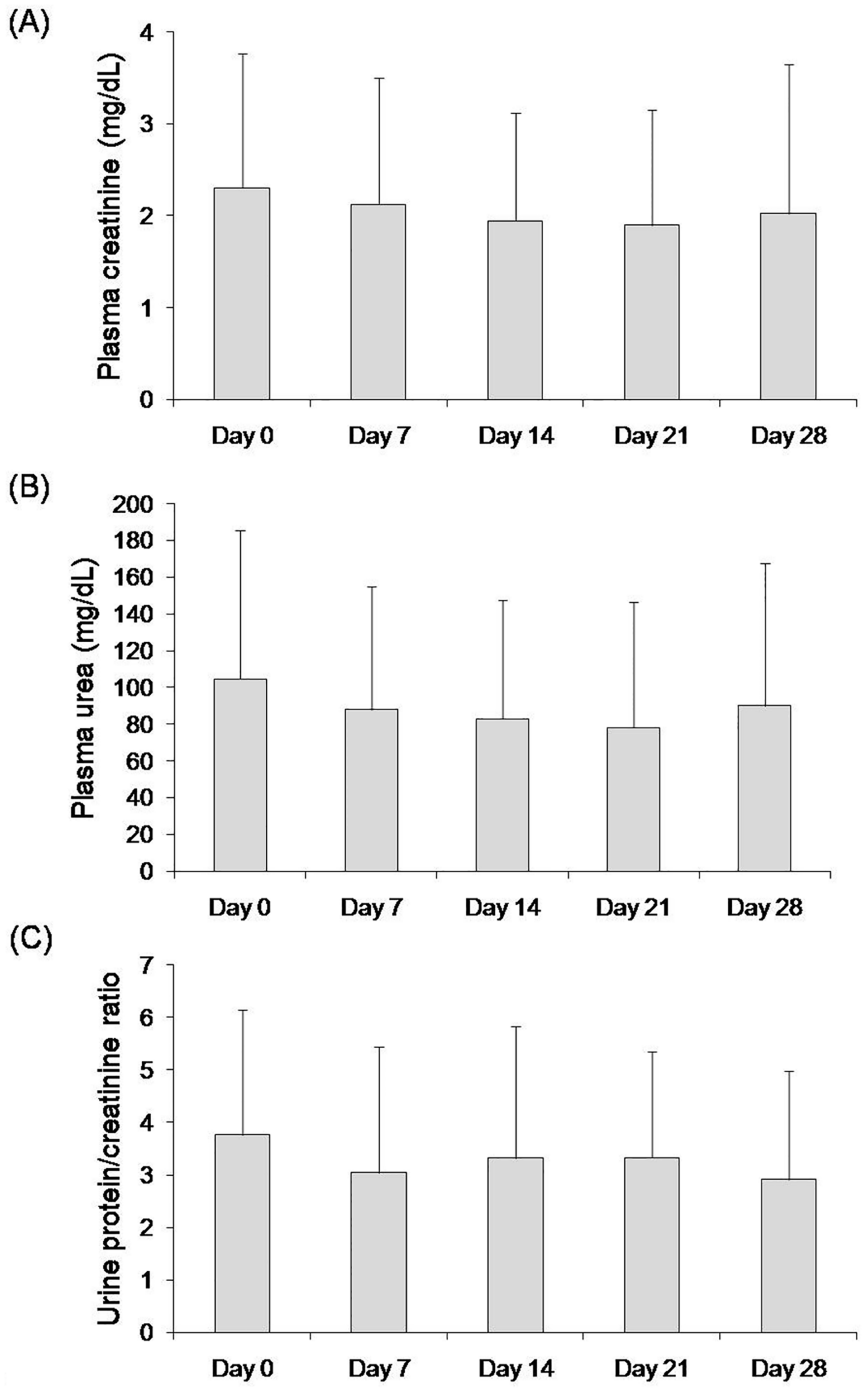
Concentrations of plasma creatinine (A), plasma urea (B) and urine protein to creatinine (UPC) ratio (C) on days 0, 7, 14, 21 and 28 of treatment with marbofloxacin in the dogs under study. Values are expressed as the mean±standard deviation.

At day 0, mean CKD stage was 2.2±1.1, with 11 dogs in stage 1, 6 dogs in stage 2, 8 dogs in stage 3, and 3 dogs in stage 4 (Table 2). Mean plasma concentrations of creatinine (2.3±1.5 mg/dL) and urea (104.5±81.2 mg/dL) were above reference ranges at day 0. Plasma creatinine did not increase during treatment, in fact it showed a non-significant tendency to decrease reaching values of 2.0±1.6 mg/dL at day 28. Plasma urea concentrations behaved similarly to creatinine. Proteinuria, UPC ≥0.5, was observed at day 0 in all dogs included in the study, with mean UPC values of 3.8±2.4. Proteinuria was attenuated during treatment and a non-significant decrease in UPC was already evident at day 7 and this tendency was maintained till day 28 (Fig 2). No significant differences in either USG (1.022 ± 0.02 vs 1.021 ± 0.01), urinary cystatin C concentration (7620 ± 11933 μg/g vs 7390 ± 11240 μg/g), or urinary ferritin (136.4±87.8 vs 154.3±75.7 μg/g) were detected when comparing day 0 and day 28.

**Table 2:** Individual renal function biomarkers of the dogs under study before (at day 0) and after (at day 28) treatment with marbofloxacin.

Dog	CKD stage	Plasma creatinine (mg/dL)		Plasma urea (mg/dL)		UPC		SBP (mmHg)		Urine Cystatin C (μg/g)		Urine Ferritin (μg/g)	
	Day 0	Day 0	Day 28	Day 0	Day 28	Day 0	Day 28	Day 0	Day 28	Day 0	Day 28	Day 0	Day 28
1	3	3.4	4.1	227.8	253.3	5.3	7.8	175	158	17830.7	38371.7	109.9	110.7
2	1	1.0	0.9	47.1	55.4	0.8	2.5	107	122	54.4	335.9	379.3	198.1
3	1	0.8	0.6	20.3	19.0	0.6	0.02	102	107	29.3	10.7	51.3	28.6
4	1	1.2	0.7	19.7	24.6	2.9	2.0	164	152	120.6	55.6	59.1	110.4
5	1	1.0	0.8	36.2	41.4	3.3	1.9	121	91	66.0	132.4	49.5	84.2
6	1	1.4	1.4	47.9	99.4	2.7	2.1	137	148	1333.4	3027.5	170.0	297.5
7	2	2.0	2.7	152.5	116.2	8.0	2.4	161	192	33998.6	25461.5	215.2	191.7
8*	4	6.6	-	359.2	-	3.0	-	126	-	-	-	-	-
9	1	1.4	0.9	61.7	36.2	6.3	3.9	177	111	45.4	53.1	207.1	268.5
10	1	1.2	0.9	49.8	43.3	0.6	0.1	117	104	711.4	23.7	92.6	53.6
11	3	3.0	3.7	241.2	250.3	6.2	3.6	172	160	4767.2	9006.3	75.4	101.9
12	2	1.9	2.0	99.8	133.1	4.1	2.6	159	155	3657.5	11810.0	113.6	125.6
13	4	5.4	4.7	301.4	365.1	4.4	2.1	186	175	21055.0	21829.2	57.9	74.3
14	2	1.8	1.1	117.7	89.2	9.3	6.1	183	173	2644.6	1624.0	164.7	221.7
15	3	2.9	4.4	136.1	182.0	5.1	3.3	178	171	1000.8	23964.7	135.5	135.7
16	3	4.9	1.5	126.8	51.55	5.8	3.4	207	209	23007.9	2560.4	139.6	196.7
17	2	1.8	0.7	79.0	39.0	5.9	5.4	209	169	18395.9	1203.9	146.5	282.0
18	1	1.0	0.6	19.4	36.4	2.8	0.2	204	180	33.4	59.7	101.0	162.4
19	1	0.9	0.7	54.2	42.9	2.7	4.9	211	230	361.0	214.8	125.4	245.7
20	3	2.5	1.6	106.4	105.9	5.2	2.9	211	231	1723.8	318.4	254.2	224.0
21	3	3.7	3.2	192.6	126.1	1.7	2.1	185	219	565.4	336.7	74.5	124.6
22	4	5.5	7.5	270.3	163.1	1.0	0.6	196	219	40058.8	34213.9	130.9	117.0
23	2	1.5	1.1	16.7	28.5	2.0	3.4	202	144	83.9	1063.9	35.2	101.4
24	3	2.3	1.7	63.8	81.5	2.5	2.1	177	161	92.1	680.5	143.8	139.3
25	2	2.0	1.6	89.2	86.6	4.4	1.7	237	193	8897.2	4841.0	82.4	211.6
26*	2	1.6	-	106.5	-	2.9	-	250	-	-	-	-	-
27	1	1.0	1.6	28.8	89.7	3.6	4.0	221	212	44.3	141.8	389.7	251.7
28	4	5.1	2.6	155.1	142.3	3.7	6.9	233	176	30808.3	14493.2	143.7	87.6
29	1	1.0	0.7	24.0	28.0	0.5	0.1	195	194	42.9	34.4	82.0	35.7
30	3	3.2	3.0	139.6	118.9	3.8	5.4	217	204	1965.8	11104.9	88.5	140.6

*: dog that did not complete the study. The results obtained from these two dogs were not included in any statistical analysis.

CKD: chronic kidney disease; SBP: systolic blood pressure; UPC: urine protein to creatinine ratio.

### Blood pressure

**Fig 3:**
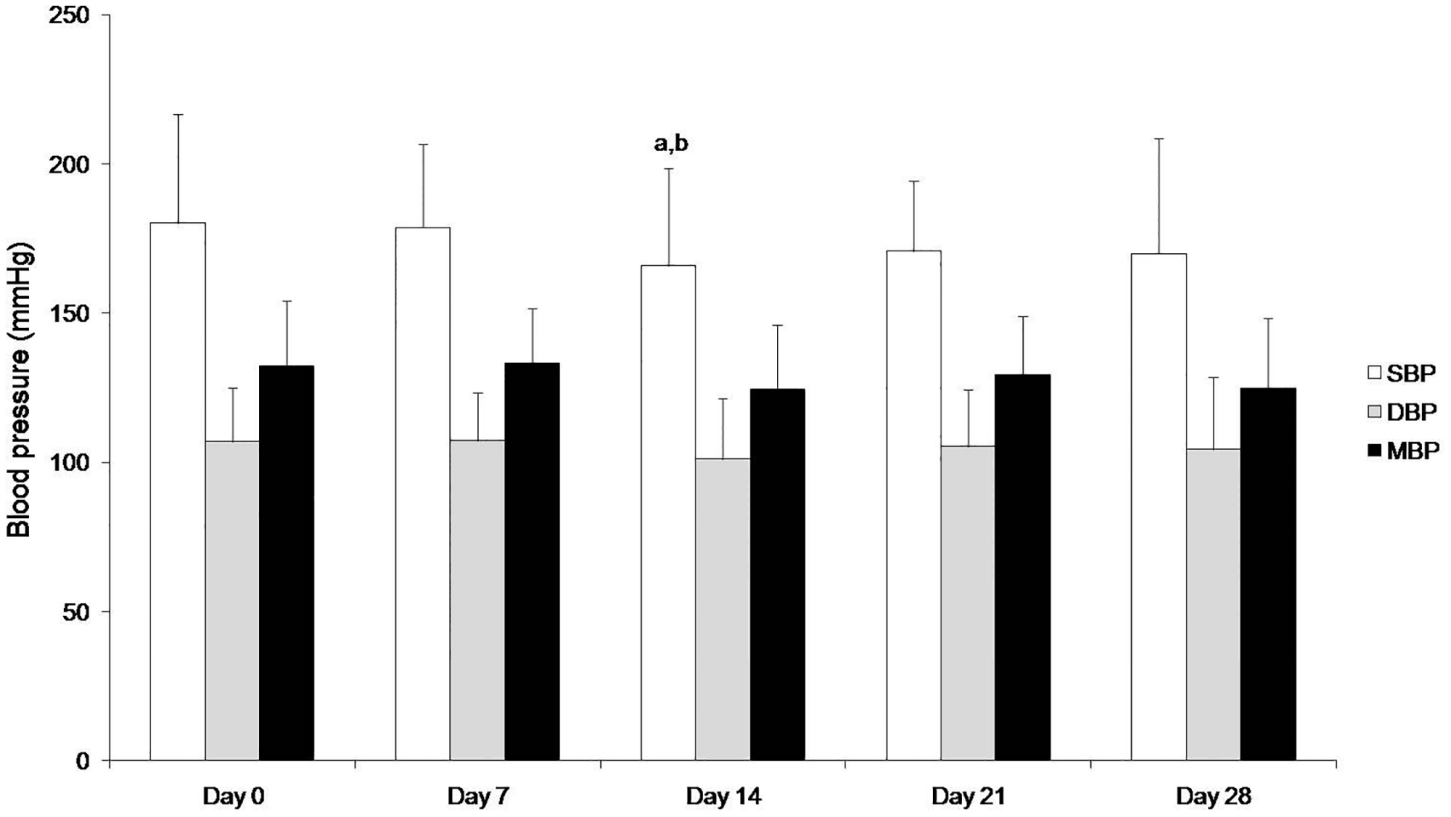
Blood pressure measurement on days 0, 7, 14, 21 and 28 of treatment with marbofloxacin in the dogs under study. Values are expressed as the mean±standard deviation. ^a^*p*<0.02 vs day 0, ^b^*p*<0.02 vs day 7.

Hypertension, as defined by IRIS (SBP >150 mmHg) was a clinical feature of the majority (82.1%) of the dogs included in the study (Table 2). As shown in Fig 3, a transient but significant decrease in blood pressure was detected up to day 14 (from 180.1±36.6 mmHg to 166.0±32.7 mmHg, *p* = 0.016). When compared with day 0, a non-significant decrease in SBP (from 180.1±36.6 mmHg to 170.0±38.8 mmHg, *p* = 0.04).

### Haematologic and biochemical parameters

The evolution of main haematological and biochemical parameters during treatment is summarized in Table 3. The mild anaemia that was detected at day 0 was not affected by treatment. Although initially dogs did not show leukocytosis (8.1±3.9 x 109/L), total leukocyte count decreased significantly (*p* = 0.006) at the end of the treatment (6.1±2.7 109/L). Treatment with marbofloxacin also resulted in attenuation of hypoalbuminemia (from 15.0±5.2 to 16.6±3.9 g/L, *p* = 0.014) and hyperglobulinemia (from to 59.0±18.1 to 54.1±18.0 g/L, *p* = 0.005). No significant changes were observed in hepatic enzymes after treatment. Marbofloxacin treatment did not influence the increased plasma phosphorus and decreased calcium levels found at day 0. Similarly, other electrolytes (Na, K, Cl, tMg) that were within normal ranges at day 0 did not change at day 28. Slight metabolic acidosis, that was not influenced by treatment with marbofloxacin, was also observed.

**Table 3:** Haematological and biochemical parameters of 28 dogs with leishmaniasis and CKD before (at day 0) and after treatment (at day 28).

	Time	
Parameters	Day 0	Day 28
**Haematology**		
Erythrocytes (10^11^/L)	5.0 ± 1.5	4.8 ± 1.4
Packed cell volume (%)	35.4 ± 10.0	33.6 ± 9.4
Haemoglobin (mg/dL)	11.5 ± 3.4	10.8 ± 3.0
Leukocytes (10^9^/L)	8.1 ± 3.9	6.1 ± 2.7^a^
Platelets count (10^11^/L)	189.2 ± 93.3	187.8 ± 85.1
**Protein profile**		
Total protein (g/L)	74.3 ± 20.3	70.7 ± 19.5^b^
Albumin (g/L)	15.0 ± 5.2	16.6 ± 3.9^b^
Globulins (g/L)	59.0 ± 18.1	54.1 ± 18.0^b^
**Liver enzymes**		
Alanine aminotransferase (IU/L)	43.5 ± 26.3	39.6 ± 31.4
Aspartate aminotransferase (IU/L)	54.0 ± 27.6	52.9 ± 29.6
Alkaline phosphatase (IU/L)	46.8 ± 30.6	41.8 ± 30.4
**Electrolytes**		
Sodium (mmol/L)	147.0 ± 6.4	144.6 ± 4.8
Potassium (mmol/L)	4.4 ± 0.5	4.6 ± 0.7
Ionized calcium (mmol/L)	1.13 ± 0.09	1.14 ± 0.09
Chloride (mmol/L)	120.6 ± 6.5	120.0 ± 6.9
Phosphate (mg/dL)	7.0 ± 3.1	7.0 ± 3.2
Total calcium (mg/dL)	8.3 ± 1.4	8.0 ± 1.6
Total magnesium (mg/dL)	2.3 ± 0.8	2.2 ± 0.6
**Acid-Base**		
pH	7.41 ± 0.07	7.42 ± 0.07
Bicarbonate (mmol/L)	20.1 ± 4.0	20.0 ± 2.6
PCO_2_ (mmHg)	32.8 ± 5.9	31.9 ± 6.4
Base Excess (mmol/L)	-3.6 ± 4.2	-3.2 ± 2.7
Anion Gap (mmol/L)	9.1 ± 9.1	9.8 ± 10.6

Values are expressed as the mean±standard deviation.

^a^ *p*<0.01 vs day 0.

^b^ *p*<0.02 vs day 0.

### Real-time PCR kinetic results

DNA isolation from lymph node aspirates was successful both pre- and post-treatment in 26 dogs. In two dogs DNA could not be obtained in both samples due to lack of sufficient tissue. In one of the dogs in which DNA isolation was successful (dog n° 28 in Table 1) parasitic DNA was not found in either pre- or post-treatment samples. This dog was maintained in the study because it showed characteristic clinical signs and a high serum IFI titer (1/640). Thus, changes in parasitic load were evaluated in 25 dogs. After treatment with marbofloxacin, the mean parasitic load decreased from 16.0±23.2 parasites/μL on day 0 to 11.3±18.7 parasites/μL on day 28, but the differences were not significant. When considered individually, parasitic load decreased in 18/25 dogs (72%) (Table 1). The reduction in parasitic load in these 18 dogs from 20.4±26.0 parasites/μL to 5.0±10.0 parasites/μL, was statistically significant (*p* = 0.018). All dogs in which parasitic load decreased also showed clinical improvement, in fact, the improvement in clinical score was more accentuated in dogs that decreased parasitic load (from 6.6±3.1 to 4.7±2.4, *p = 0*.01) than in dogs that did not (in which the clinical score was reduced from 7.1±3.8 to 6.1±4.2, *p* = 0.111). Therefore, according to the efficacy criteria previously set, the treatment was efficacious in 18 dogs (72% of the population).

### Correlations

At day 0 the following significant correlations were found: Parasitic load was correlated with UPC (r = -0.516, *p* = 0.008) and with globulins (r = 0.559, *p* = 0.004). Albumin was correlated with UPC (r = -0.623, *p*<0.001) and with DBP (r = -0.485, *p* = 0.009). The UPC was correlated with DBP (r = 0.572, *p* = 0.001) and with MBP (r = 0.533, *p* = 0.003). Antibody titers correlated with renal parameters: urea (r = 0.681, *p*<0.001) and creatinine (r = 0.648, *p*<0.001). Urine cystatin C had very good correlation with traditional markers of renal function: urea (r = 0.662, *p*<0.001), creatinine (r = 0.696, *p*<0.001) and phosphate (r = 0.619, *p*<0.001).

At day 28 the following significant correlations were found: Parasitic load was correlated with packed cell volume (r = -0.548, *p* = 0.005) and with DBP (r = 0.548, *p* = 0.005). Albumin was well correlated with urine ferritin (r = -0.629, *p*<0.001) and with DBP (r = -0.468, *p* = 0.01). The UPC maintained correlation with albumin, but weaker (r = -0.402, *p* = 0.03) than at time 0, and with total proteins (r = 0.590, *p* = 0.001). Antibody titers correlated very well with renal parameters: urea (r = 0.638, *p*<0.001), creatinine (r = 0.609, *p* = 0.001) and cystatin C (r = 0.559, *p* = 0.002). Urine cystatin C, as in day 0, had very good correlations with traditional renal parameters: urea (r = 0.725, *p*<0.001), creatinine (r = 0.846, p<0.001) and phosphate (r = 0.765, *p*<0.001). However, urine ferritin was not correlated with renal parameters, but showed a good correlation with total proteins (r = -0.513, *p* = 0.005), albumin (r = -0.629, *p*<0.001) and DBP (r = 0.480, *p* = 0.01). A strong correlation between Anion Gap and SBP (r = 0.696, *p* = 0.003) was also found.

**Fig 4:**
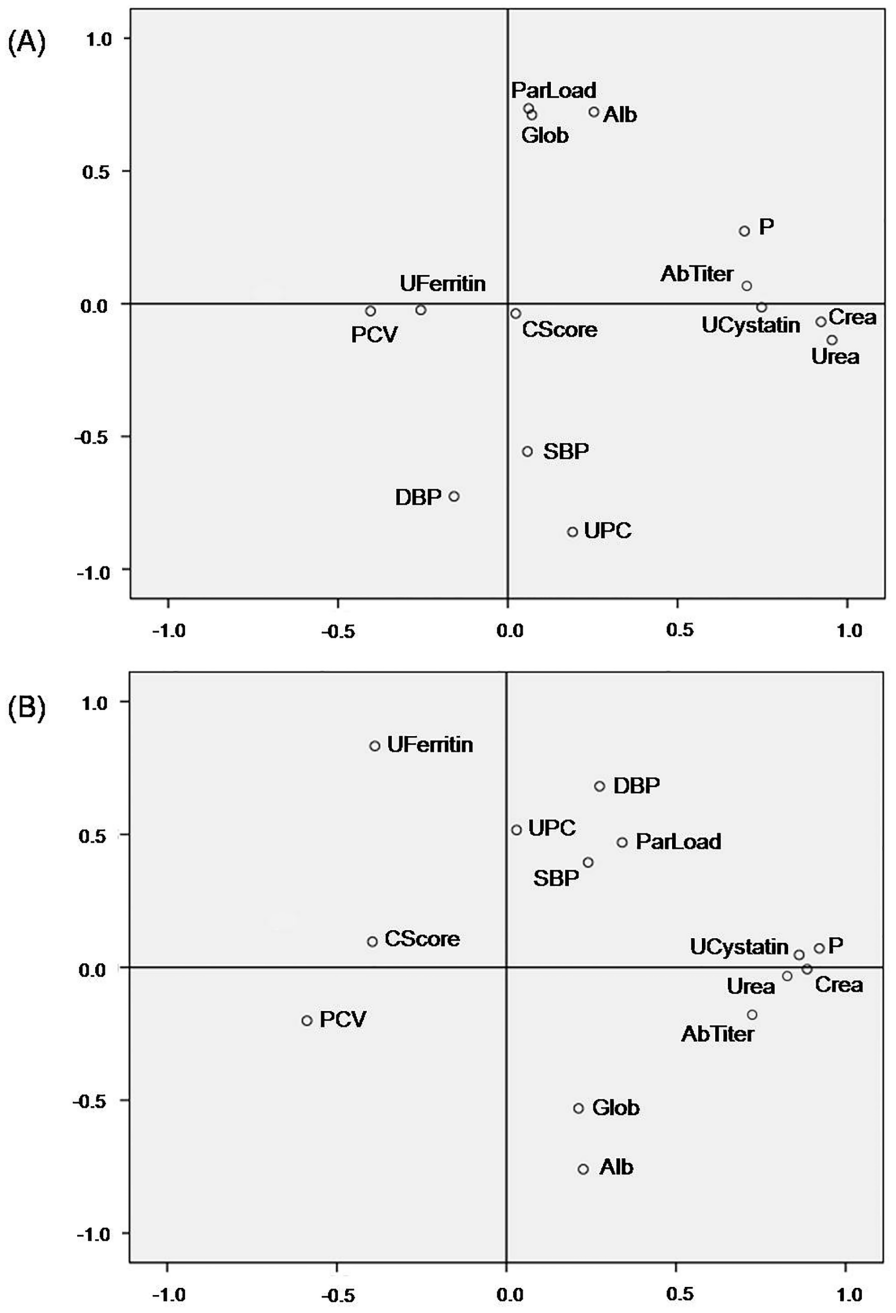
Principal components analysis plot. **Interrelation between the main parameters studied at day 0 (A) and at day 28 (B)**. Alb: albumin; AbTiter: antibody titer; Crea: creatinine; CScore: clinical score; DBP: diastolic blood pressure; Glob: globulins; P: phosphate; ParLoad: parasitic load; PCV: packed cell volume; SBP: systolic blood pressure; UCystatin: urine cystatin C; UFerritin: urine ferritin; UPC: urine protein to creatinine ratio.

The interrelation between the main variables studied is graphically shown in a principal components analysis plot (Fig 4).

## Discussion

This study was aimed at investigating the safety and efficacy of treatment with marbofloxacin in dogs affected by leishmaniasis that had decreased renal function. The results show that marbofloxacin is a safe and effective drug that does not aggravate renal disease in dogs with leishmaniasis.

The efficacy of marbofloxacin for treatment of leishmaniasis has been previously evaluated in dogs with intact renal function. Rougier et al. (2012) [18] showed a clinical improvement comparable with what we have found after 28 days of treatment. It is important to note that, theoretically, the response to treatment should not be as good in dogs with renal disease since they usually show progressive signs related to organ damage (e.g. gastrointestinal signs) which do not necessarily improve after eliminating the parasite [15]. The response to treatment was not correlated with the CKD stage, suggesting that marbofloxacin is likely to be effective even in dogs with severe renal disease, which is the subpopulation of dogs with leishmaniasis in which other treatments may be contraindicated. In addition to the anti-leishmanial actions, the antibiotic effect of marbofloxacin could have contributed to improvement of clinical signs in which secondary bacterial infections may play a role (e.g. dermatologic signs). In our study we only evaluated the dogs during and at the end of the treatment with marbofloxacin because, for ethical reasons, after day 28 all dogs started another treatments (allopurinol and different drugs for CKD); thus, from this time on it was not possible to isolate the effect of marbofloxacin. However, as shown by Rougier et al. (2012) [18], clinical signs keep improving for several months after finishing treatment with marbofloxacin.

The efficacy of the treatment was assessed by both clinical improvement and decrease in parasitic load. The PCR results showed a decrease in parasitic load in most dogs but the drug was not able to completely eliminate the parasite from lymph nodes. In addition, there was a subpopulation of dogs in which parasitic load did not decrease. It is interesting to note that the improvement in clinical score was more accentuated in dogs that decreased parasitic load than in dogs that did not. Evidence for a relationship between parasitic load and clinical manifestations has been previously reported in dogs with leishmaniasis [25]. However, a lack of correlation between changes in clinical signs and parasitic load has also been reported [26–28], and it is known that anti-leishmanial treatments do not eliminate all parasites [27, 29–35]. Serological CanL titers were not repeated after treatment because, although they have been recently reported as potentially useful shortly after treatment [36], data in the literature support that a minimum period of 6 months is required to find significant changes in IFI results [5]. Thus most authors consider that serology cannot be used as a reliable criterion in the short-time follow-up of the clinical evolution of treated dogs [5, 33].

Renal disease secondary to immune complex deposition is very common in dogs with leishmaniasis. Renal damage usually starts at the glomerulus and then progress to affect the entire nephron. The clinical pathology profile in the dogs of this study (proteinuria, azotemia, increased urine ferritin) points to a predominance of glomerular dysfunction, although some tubular dysfunction was also present as evidenced by the increase in urinary cystatin C. The prevalence of renal damage in CanL is very high and it is estimated that about 50% of dogs with leishmaniasis show changes in clinical pathology characteristic of renal disease. Since most effective therapies for treatment of CanL are known to induce renal injury, there is a need for effective treatments that preserve renal function. Marbofloxacin has been shown to be safe in dogs with normal renal function. The main objective of this study was to evaluate with detail the renal changes induced by treatment with marbofloxacin in a population of dogs with CKD stages 1–4. Marbofloxacin treatment did not induce further renal damage in the dogs of this study since renal parameters did not show impairment after administering marbofloxacin for 28 days.

A non-significant decrease in the urinary loss of proteins, which was more accentuated in dogs with higher baseline values of UPC, was detected in the course of the treatment. A reduction in proteinuria in dogs with leishmaniasis has been previously reported after treatment with glucantime [37] and with allopurinol [14]. The time frame for the decrease in protein loss by urine is similar after treatment with marbofloxacin and glucantime and much shorter than after treatment with allopurinol. The reduction in systemic blood pressure observed after treatment may also have contributed to the decrease in protein loss by urine. In our study a strong correlation was found between blood pressure and UPC. These data are in agreement with previous reports linking hypertension with proteinuria in dogs with leishmaniasis [7, 38–40].

Systemic hypertension is a common finding in dogs with renal disease secondary to leishmaniasis [7]. In humans, hypertension may appear early in the course of renal disease and has been reported not to be directly correlated with plasma creatinine concentrations [41]. In our study, there was a weak correlation between plasma creatinine and SBP at the beginning (day 0) but not at the end (day 28) of the study. The lack of correlation at the end of the study may reflect the fact that treatment resulted in more effective decrease in blood pressure than in plasma creatinine. In any case, the decline in blood pressure observed after treatment with marbofloxacin is an indication of renal improvement. The decrease in blood pressure found in the present study would reduce the risk of organ damage from very high to moderate (IRIS staging for CKD).

The lack of deleterious effect of marbofloxacin on renal function is supported by the fact that plasma concentrations of creatinine and urea did not increase during treatment. The tendency to reduced azotemia detected in the study could be related to the general improvement after treatment which would result in better hydration of the dogs. Plasma urea and creatinine correlated with antibody titers but not with PCR results at day 0, suggesting that renal damage is more linked to the host immune response than to the actual parasitic load.

An increase in plasma proteins secondary to hyperglobulinemia and a decrease in plasma albumin, that result in a reduced A/G ratio, are common findings in dogs with leishmaniasis. Parasitic load showed significant correlation with plasma globulins. After treatment with marbofloxacin a significant increase in the A/G ratio was observed. The increase in the A/G ratio was due to both a decrease in globulins, secondary to decreased parasitic stimulation of the immune system, and to an increase in albumin. In dogs with leishmaniasis hypoalbuminemia is due to renal loss, decreased synthesis secondary to chronic inflammation and decreased protein intake [34]. In agreement with Pierantozzi et al. (2013) [37], at day 0 a significant inverse correlation was found between UPC and plasma albumin, suggesting that renal loss was a major factor determining hypoalbuminemia. As previously reported [18, 42] plasma albumin concentration increased after treatment. Thus, the accumulated reduction in UPC may have played an important role in the increase in albumin after treatment.

The toxicity of drugs used for the treatment of CanL is well known and this is one of the major limitations when using these treatments in dogs with reduced renal function. Both meglumine antimoniate and miltefosine have been reported to cause gastrointestinal side effects (vomiting, diarrhoea, anorexia) [9, 10, 12, 13]. In addition, antimonials are highly nephrotoxic and dogs treated with glucantime have been reported to suffer severe tubular damage (tubular cell apoptosis and necrosis) [11]. Renal toxicity has also been reported after treatment with miltefosine in humans [43, 44]. Treatment with marbofloxacin was very well tolerated and only two dogs showed minor self-limiting episodes of diarrhoea. In addition to the lack of deleterious actions on the kidneys, and in contrast to antimonials [34], marbofloxacin did not increase plasma concentrations of liver enzymes. Moreover, no significant changes were detected after treatment in any of the multiple biochemical parameters under study.

In conclusion, the results of this study demonstrate that, in addition to be effective for treatment of CanL, marbofloxacin is a very safe drug in dogs with CKD and leishmaniasis.
